# Supplementary material for: Gestational toxoplasmosis treatment changes the child’s prognosis: A cohort study in southern Brazil
Source: PLoS Negl Trop Dis. 2023 Sep 29;17(9):e0011544. doi: 10.1371/journal.pntd.0011544 (PMC10593203; doi:10.1371/journal.pntd.0011544)
Supplement: S1 File — (DOCX) [file pntd.0011544.s001.DOCX]

| **MATERNITY WARD** |
| --- |
| Anthropometric data at birth |
| - Weight - Head circumference |
| Blood and radiological exams collected from newborns after 24 hours of life |
| - Complete blood count (CBC) |
| - ALT: alanine transaminase |
| - AST: aspartate transaminase |
| - CSF: cerebrospinal fluid |
| - Serology anti – *T. gondii* IgM and IgG |
| - Cranial tomography |
| Antibiotic treatment |
| - Sulfadiazine 50 mg/kg 12/12h + |
| - Pyrimethamine 1 mg/kg/day for 2 months, then 1 mg/kg/day every other day + |
| - Folinic acid 2mg every other day |
| The pyrimethamine, sulfadiazine and folinic acid capsules are prepared by the hospital pharmacy according to the weight of the infant. |
| # At discharge, we deliver sufficient medication to the family until the Basic Health Unit provides the medication. All data is noted in the child's wallet. Before discharge, the family schedules the ToxoPed outpatient clinic, consultation with an ophthalmologist, evaluation with a speech therapist and exams to be collected by the HUM laboratory within 20 -30 days of life. |
| **First appointment in “ToxoPed” clinic** |
| - With 30–45 days of life |
| - We check the exams from birth and after discharge (CBC, ALT, AST, Serology anti- *T. gondii* IgM and IgG) |
| - If there are no adverse drug effects, the antibiotic treatment is maintained |
| - We request CBC, ALT, AST and anti-*T. gondii* IgM and IgG serology |
| - The next return will be in 45 days ( the child will be nearly 90 days old) |
| **Second appointment in “ToxoPed” clinic** |
| - Within 90 days of life |
| - We check the exams requested before (CBC, ALT, AST and anti-*T. gondii* IgM and IgG serology) |
| - If the IgG serology decreases compared to the two previous ones, the medication is suspended |
| - We request anti-*T. gondii* IgM and IgG serology |
| - The return will be in two months |
|  |
| **Third appointment in “ToxoPed” clinic** |
|  |
| - From here, if the serology continues to decline, the child remains without medication and the next return will be every 3 months with a new IgG and IgM serology. Anti-*T. gondii* serology is requested until we observe the negative value of IgG |
| **Infected children** |
| Children considered infected continued anti-toxoplasma medication until 12 months of life. After completing the first year of life, the medication was discontinued |
| - The return is every 2 months or before if there are some adverse antibiotic effects |
| - CBC, ALT, AST and anti-*T. gondii* IgM and IgG serology are checked in all returns |
| Ophthalmologic evaluation every 6 months until two years and then once a year |
| Phonoaudiological evaluation with BERA is requested for all infected children |

**Supplementery Table A. Protocol used in University Hospital of Maringá**

**Supplementary Table B. Risk Relative, congenital toxoplasmosis X mother treatment**

|  | Infected | Exposed |
| --- | --- | --- |
| Mtyes | 4 (36%) | 44 (88%) |
| Mtno | 7 (64%) | 6 (12%) |
| risk ratio 0.15 with 95% C.I.( 0.05-0.45) p = 0.0006  P value were calculated by Fisher- test  Mtno: no mother treatment, Mtyes: yes mother treatment | | |

**Supplementary Table C. Risk Relative, neutropenia X congenital toxoplasmosis**

|  | Nyes | Nno |
| --- | --- | --- |
| Exposed | 13 (27%) | 35 (73%) |
| infected | 8 (73%) | 3 (27%) |
| risk ratio 0.37 with 95% C.I. (0.20-0.67) p = 0.0010  P value were calculated by Fisher- test  Nno: no neutropenia, Nyes: yes neutropenia | | |

**Supplementary Table D. Logist regression, neutropenia X pyrimethamine**

| Logistic regression | log(OR)^1^ | 95% CI^1^ | p-value^1^ |
| --- | --- | --- | --- |
| mother_treatment |  |  |  |
| E | - | - | - |
| PSAE | 0.64 | -0.71 - 2.0 | 0.4 |
| NT | 1.9 | 0.29 - 3.4 | 0.020 |
| ^1^ OR = Odds Ratio, CI = Confidence Interval  E = spiramicyn, PSAE = pyrimethamine + sulfadiazine + folinic acid + spiramicyn, NT=no treatment | | | |

**Supplementary Table E. Dimensions extracted by Factor Analysis of Mixed Data (FAMD)**

|  | Dim.1 | Dim.2 | Dim.3 | Dim.4 | Dim.5 |
| --- | --- | --- | --- | --- | --- |
| Variance Eigenvalues | 2.890 | 2.091 | 1.775 | 1.099 | 1.000 |
| % of var. | 26.275 | 19.010 | 16.137 | 9.994 | 9.090 |
| Cumulative % of var. | 26.275 | 45.285 | 61.422 | 71.417 | 80.506 |
| The first two dimensions (Dim) of FAMD accounted for 45.28% of the total inertia of the described/dimension-reduced data frame. | | | | | |

**Supplementary Table F. Categories**

|  | Dim.1 | ctr | cos2 | v.test | p-value | Dim.2 | ctr | cos2 | v.test | p-value | Dim.3 | ctr | cos2 | v.test |
| --- | --- | --- | --- | --- | --- | --- | --- | --- | --- | --- | --- | --- | --- | --- |
| 1t | -0.178 | 0.087 | 0.008 | -0.443 |  | -0.996 | 5.208 | 0.240 | -2.912 |  | 0.781 | 4.446 | 0.148 | 2.479 |
| 2t | -0.962 | 4.179 | 0.425 | -3.411 |  | 0.069 | 0.041 | 0.002 | 0.287 |  | 0.085 | 0.086 | 0.003 | 0.383 |
| 3t | -0.389 | 0.416 | 0.043 | -0.968 |  | 0.013 | 0.001 | 0.000 | 0.037 |  | -0.361 | 0.952 | 0.037 | -1.147 |
| maternity | 3.008 | 17.751 | 0.777 | 6.068 | 8.546 e-14 | 1.219 | 5.567 | 0.128 | 2.890 |  | -0.782 | 3.184 | 0.053 | -2.014 |
| Mtno | 2.438 | 15.168 | 0.747 | 5.782 | 5.027 e-12 | 0.941 | 4.315 | 0.111 | 2.623 |  | -0.813 | 4.469 | 0.083 | -2.460 |
| MTyes | -0.660 | 4.108 | 0.747 | -5.782 |  | -0.255 | 1.169 | 0.111 | -2.623 | 0.0075 | 0.220 | 1.210 | 0.083 | 2.460 |
| exposed | -0.565 | 3.127 | 0.761 | -5.484 |  | -0.193 | 0.701 | 0.089 | -2.209 | 0.0259 | 0.099 | 0.256 | 0.023 | 1.229 |
| infected | 2.566 | 14.216 | 0.761 | 5.484 |  | 0.879 | 3.187 | 0.089 | 2.209 |  | -0.451 | 1.163 | 0.023 | -1.229 |
| Nno | -0.541 | 2.298 | 0.402 | -3.402 |  | -0.250 | 0.935 | 0.086 | -1.846 | 3.98e-04 | 0.211 | 0.929 | 0.061 | 1.695 |
| Nyes | 1.031 | 4.376 | 0.402 | 3.402 |  | 0.476 | 1.781 | 0.086 | 1.846 |  | -0.402 | 1.770 | 0.061 | -1.695 |
| Moment of gestational toxoplasmosis diagnostic = 1t: first trimester, 2t: second trimester, 3t: thirst trimester, maternity  Antibiotic mother treatment = Mtno: no mother treatment, MTyes: yes mother treatment  Congenital toxoplasmosis = exposed, infected  Neutropenia = Nno: no neutropenia, Nyes: yes neutropenia | | | | | | | | | | | | | | |

**Supplementery Table G. Quantitative variables**

|  | Dim.1 | ctr | cos2 | p-value | Dim.2 | ctr | cos2 | p-value | Dim.3 | ctr | cos2 |
| --- | --- | --- | --- | --- | --- | --- | --- | --- | --- | --- | --- |
| time_motherdiagnosis_ treatment | 0.591 | 12.099 | 0.350 | 5.214e-07 | -0.173 | 1.429 | 0.030 |  | 0.770 | 33.414 | 0.593 |
| gestational_age_birth | 0.070 | 0.169 | 0.005 |  | 0.410 | 8.035 | 0.168 |  | 0.082 | 0.380 | 0.007 |
| protein_lcr | 0.624 | 13.479 | 0.390 | 7.669e-08 | -0.260 | 3.224 | 0.067 |  | 0.718 | 29.033 | 0.515 |
| weigth_birth | -0.382 | 5.057 | 0.146 |  | 0.828 | 32.805 | 0.686 | 1.794e-16 | 0.360 | 7.303 | 0.130 |
| head_circunference_  birth | -0.317 | 3.469 | 0.100 |  | 0.813 | 31.605 | 0.661 | 1.764e-15 | 0.450 | 11.405 | 0.202 |
| time_motherdiagnosis_ treatment = time between the mother diagnosis and mother treatement  protein_lcr = protein in cerebrospinal fluid | | | | | | | | | | | |

**Supplementary Figure A. FAMD Quantitative variables**


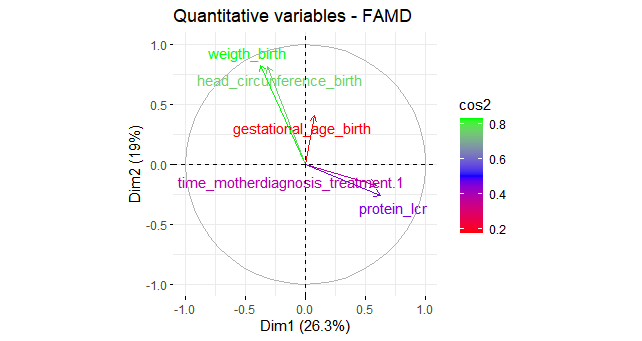


Moment of gestational toxoplasmosis diagnostic = 1t: first trimester, 2t: second trimester, 3t: thirst trimester, maternity

Antibiotic mother treatment = Mtno: no mother treatment, MTyes: yes mother treatment

Congenital toxoplasmosis = exposed, infected

Neutropenia = Nno: no neutropenia, Nyes: yes neutropenia

protein_lcr = protein in cerebrospinal fluid

time_motherdiagnosis_treatment.1 = = time_motherdiagnosis_ treatment = time between the mother diagnosis and mother treatemen

Explanation: In this figure, we observe quantitative variables' distribution and contributions in dimensions one and two (Dim 1 and Dim 2), the more significant the cos2, the more outstanding the contribution.

**Supplementary Figure B. FAMD Qualitative variables**


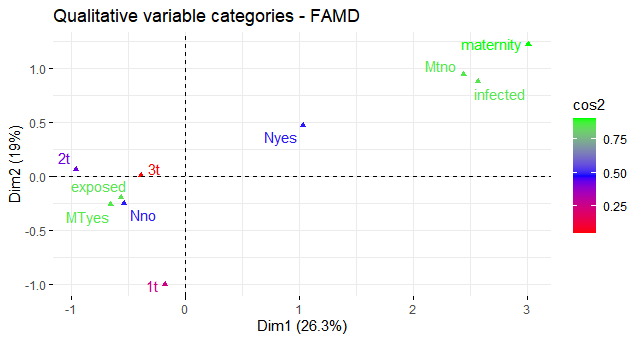


Moment of gestational toxoplasmosis diagnostic = 1t: first trimester, 2t: second trimester, 3t: thirst trimester, maternity

Antibiotic mother treatment = Mtno: no mother treatment, MTyes: yes mother treatment

Congenital toxoplasmosis = exposed, infected

Neutropenia = Nno: no neutropenia, Nyes: yes neutropenia

Explanation: We can observe the formation of two groups; in dimension 1 (Dim1), the variables "no maternal treatment" (Mno), "maternity", "Neutropenia yes" (Nyes) group the infected individuals. In dimension 2 (Dim2), there is a closeness between the variables "second trimester" (2t), “third trimester” (3t), “first trimester” (1t), and the exposed individuals. The higher the co2, the more significant the variable's contribution.

**Supplementary Figure C. FAMD Qualitative, Quantitative and Individual map**


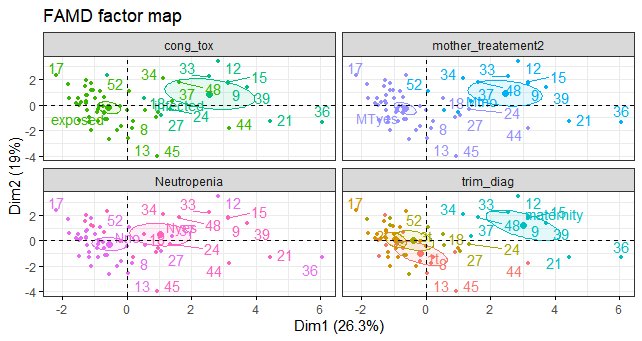


Moment of gestational toxoplasmosis diagnostic = 1t: first trimester, 2t: second trimester, 3t: thirst trimester, maternity

Antibiotic mother treatment = Mtno: no mother treatment, MTyes: yes mother treatment

Congenital toxoplasmosis = exposed, infected

Neutropenia = Nno: no neutropenia, Nyes: yes neutropenia

Explanation: In this figure, we observe the spatial distribution of individuals according to the variables studied

**Suplemmentary Figure D. Decision tree**


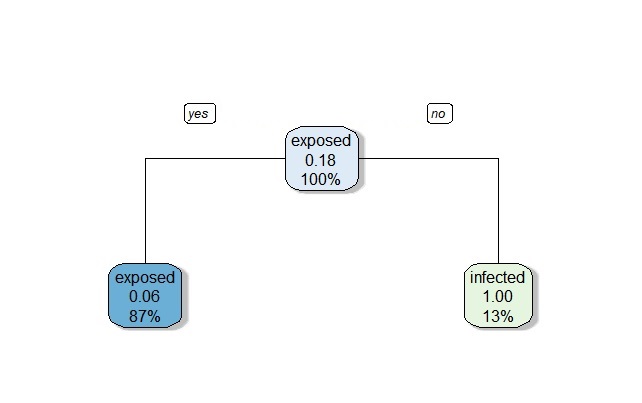


Three decreasing IgG anti *T-gondii*

Explanation: This figure shows three decreases in the anti-*T. gondii* serology IgG is an adequate resource for deciding whether to discontinue drug treatment because there is 87% chance that the choice will fall on exposed, not infected individuals.
