# Supplementary material for: Gestational toxoplasmosis treatment changes the child’s prognosis: A cohort study in southern Brazil
Source: PLoS Negl Trop Dis. 2023 Sep 29;17(9):e0011544. doi: 10.1371/journal.pntd.0011544 (PMC10593203; doi:10.1371/journal.pntd.0011544)
Supplement: Attachment — Submitted filename: answer_Reviewer_PLOS.docx [file pntd.0011544.s004.docx]

GENERAL COMMENTS BY THE AUTHORS –

We would like to thank the reviewers for their insight and thoughtful critique of our manuscript. We believe that we have fully addressed all of these concerns and comments, which will increase the overall impact of the manuscript.

RESPONSE TO REVIEWER 1

1. In the field of sample size and compliance with ethical requirements, there is a little ambiguity, which should be given more comprehensive explanations and evidence by the authors.

AUTHORS: Thank you for your consideration. There was a change in the codes initially cited as we unified two research projects, and a new number regulation was generated after the article was submitted. We fixed the detected inconsistency.

Lines 141-142: “The Ethics and Research Committee of the State University of Maringa approved this study (CAAE Nº 09286918.5.0000.0104).”

2. Toxoplasma gondii should be written in italics throughout the manuscript.
AUTHORS: Thank you for your observation. We provide necessary fixes.

RESPONSE TO REVIEWER 2:

1. The authors need to include the information on how the serological tests to detect IgG and IgM antibodies to T. gondii were performed? Did they use commercial kits for this purpose? If this is the case, they need to provide name of the kits and name of the companies that provide the kits.

AUTHORS: Thank you for your observation. We added the information requested.

Lines 214-219: Between 2017 - 2018, the amplified chemiluminescent method was used with Vitros ECiQ Toxoplasma IgG and IgM assays (Ortho-Clinical Diagnostics©), according to the manufacturer’s specifications. Since December 2019, we have used microparticle immunoassay with ARCHITECT Toxoplasma IgG and IgM kits, according to the manufacturer’s specifications (Abbott©, Wiesbaden, Germany).

2. The first paragraph of the Results section (Page 11): The authors state “All children started drug treatment in the maternity ward and 52/61 were suspended after three reducing serological tests (anti-T. gondii IgG)”. This statement makes readers to consider that 9 (61-52 =9) children showed serological evidence for having congenital infection. The authors also state “Among the eleven infected children, three were detected after discontinuing the medications”. These statements together make readers to consider 12 (9 + 3= 12) children were congenitally infected. However, the actual number of infected children was 11. The authors need to provide additional information to explain these numbers.

AUTHORS: Thank you for your observation. We provide necessary fixes.

Lines 280-285: ”All children started drug treatment in the maternity ward and 53/61 were suspended after three reducing serological tests (anti-*T. gondii* IgG). Among the eleven infected children, three were detected after discontinuing the medications; their anti-*T. gondii* IgG increased and the anti-*T. gondii.*”

3. In relation to neutropenia in congenitally infected children, the authors referred an article an occurrence of neutropenia in a murine model of acute T. gondii infection. The authors need to include the information that this murine model is a lethal infection of animals with the virulent RH strain of the parasite. The authors also need to be more careful about applying the information from this murine model of the lethal infection to congenitally infected children.

AUTHORS: Thank you for your observation. We made changes to clear the text.

Lines 341-342: “An experimental study showed that mice infected by *T. gondii* (RH strain) presented leukocyte depletion in the bone marrow and peripheral blood “

Taking into account your last recommendation.” The authors also need to be more careful about”, we rewrote the text.

Lines 344-346: “In this way, we postulated that the parasite, itself, might cause a higher risk of neutropenia in infected children. However, further human studies are needed to prove this point of view”

It is important to point out that although we looked for studies that evaluated neutrophils, we only found their justification through the use of antiparasitic drugs. Unfortunately we did not find studies with neutropenia analysis and bone marrow evaluation in humans. Generally, studies focus more on the lymphocyte component and its variation between infected and exposed patients.

4. Among  11 congenitally infected children, 5 were asymptomatic. It is important to provide the information on whether treatment of their mothers makes any association to determine their infected children become symptomatic or asymptomatic.

AUTHORS: Thank you for your observation.

Lines 290-293: “Among the asymptomatics, three mothers were diagnosed in the maternity ward, so they did not receive any drug treatment. The other two mothers were diagnosed in the third trimester and started medication four and seven weeks from diagnosis.

5. It is also important to perform the analysis on whether neutropenia is associated with symptomatic infection or not.

AUTHORS: Thank you for your observation. We made changes to clear the text.

Lines 280-285: “All children who had neutropenia were under outpatient follow-up and did not have other diseases that could cause a low number of neutrophils. No neutropenic child was hospitalized when the blood test was altered. Among the infected patients who developed neutropenia, three were asymptomatic, and five were symptomatic (1 with ocular lesions and brain calcifications, 2 with brain calcifications, and 2 with ocular lesions).”

6. The authors need to provide more detailed descriptions in the description in the last paragraph of the “Results” section. This description needs to include what each of Figs. 2a, 2b, and 2c indicates and how these figures need to be read. It is also helpful to explain what each of Supplemental Figures 1, 2, and 3 indicates.

AUTHORS: Thank you for your observation. We made changes to clear the text

Lines 233-244: “We used factor analysis of mixed data (FAMD) to analyze the data set. FAMD is a principal components method to describe, summarizes and visualize multidimensional matrix with mixed data. As any principal components method, its aim is to study the similarities between individuals, the relationships between variables and to link the study of the individuals with the variables. Such methods reduce the dimensionality of the data and provide subspace that best represent the data. Briefly, this statistical method makes possible to analyze the similarity between individuals by considering mixed types of variables. We explore the association between all variables (S Tables 5 and 6). In the multifactorial evaluation, the quantitative variables were grouped into dimensions 1 and 2 according to their contribution value (S Fig 1 and 2).”

Lines 296-303: “In figure 2a, we can observe the formation of two groups; in dimension 1 (Dim1), the variables "no maternal treatment" (Mno), "maternity", "Neutropenia yes" (Nyes) group the infected individuals. In dimension 2 (Dim2), there is a closeness between the variables "second trimester" (2t), “third trimester” (3t), “first trimester” (1t), and the exposed individuals. In figure 2b, the exposed and infected groups are well characterized visually. In this figure, each individual is represented by a dot. In Figure 2c, we visualize the individuals grouped by the variables that describe them.”

Lines 305-311: Figure 2. Correlation among quantitative and qualitative variables. 2a: the variables with the highest correlation are closer to each other, we can observe a group (Dim 1) formed by the infected children (infected), untreated mothers (Mtno) and the diagnosis performed at the hospital maternity (maternity). The other group (Dim 2) includes children who have not confirmed congenital infection (exposed), maternal treatment (MTyes), children without neutropenia (Nno) and diagnoses performed in the second, third and first trimesters (1t, 2t, 3t). In figure 2b, the individuals are represented by dots. In 2c we can visualize individuals grouped by the variables

“reviewer…explain what each of Supplemental Figures 1, 2, and 3 indicates”

AUTHORS: S Figure 1: “In this figure, we observe quantitative variables' distribution and contributions in dimensions one and two (Dim 1 and Dim 2), the more significant the cos2, the more outstanding the contribution.”

S Figure 2: “We can observe the formation of two groups; in dimension 1 (Dim1), the variables "no maternal treatment" (Mno), "maternity", "Neutropenia yes" (Nyes) group the infected individuals. In dimension 2 (Dim2), there is a closeness between the variables "second trimester" (2t), “third trimester” (3t), “first trimester” (1t), and the exposed individuals. The higher the co2, the more significant the variable's contribution.”

S Figure 3: “In this figure, we observe the spatial distribution of individuals according to the variables studied.”

7. The end of the first paragraph on page 16: The meaning the following sentence is unclear; “Bearing this in mind and given the data that point to the presence of more virulent strains in Brazil (greater chance of tissue damage in infected children), the protocol is to discharge all children born to a mother with acute toxoplasmosis with triple treatment (SPF).”

AUTHORS: Thank you for the suggestion. We rewrote the sentence.

Lines 359-362: “Bearing this and given the presence of more virulent strains in Brazil, that may cause tissue damage in infected children, our protocol is to discharge all children born to a mother with acute toxoplasmosis with triple treatment (SPF).”

8. In Supplemental Tables, all abbreviations (e.g. MTyes) need to be spelled out at the bottom of each Table.

AUTHORS: Thank you . We added the meaning of the abbreviation in S table 3.

9. It is unclear what Supplemental Figure 4 indicates.

AUTHORS: We added the explanation : “This figure shows three decreases in the anti-T. gondii serology IgG is an adequate resource for deciding whether to discontinue drug treatment because there is 87% chance that the choice will fall on exposed, not infected individuals.”

10. It will be helpful to include the information on what genotypes of T. gondii are abundant in the southern Brazil, where this clinical study was performed.

AUTHORS: Indeed, this information would be of great value. However, we do not have many studies on genotypes found in humans in the studied region. Thus, we added the genotypes most commonly found in Brazil that have already been associated with the disease in children with congenital infection.

Lines 67-71: In Brazilian studies that identified the strain of T. gondii in children with congenital toxoplasmosis, it was possible to isolate fifteen different genotypes - #11 (BrII), #8 (BrIII), and the atypical #36, #41, #67, #108, #162, #166, #206, #207, #208, #209, #210, #211, #212 [6].#36, #41, #67, #108, #162, #166, #206, #207, #208, #209, #210, #211, #212

RESPONSE TO REVIEWER 3 (e-mail):

”…the manuscript needs to be carefully checked in terms of writing (for example: line 219 has a dot added after the parentheses and should be deleted) and the sentences need to be strengthened in terms of academic writing.”

AUTHORS: Thank you. We rewrote the sentence. Between 2017 - 2018, the amplified chemiluminescent method was used with Vitros ECiQ Toxoplasma IgG and IgM assays (Ortho-Clinical Diagnostics©), according to the manufacturer’s specifications. Since December 2019, we have used microparticle immunoassay with ARCHITECT Toxoplasma IgG and IgM kits, according to the manufacturer’s specifications (Abbott©, Wiesbaden, Germany).
